# Individuals With Hemiparetic Stroke Accurately Match Torques They Generate About Each Elbow Joint

**DOI:** 10.3389/fnins.2019.01293

**Published:** 2019-11-28

**Authors:** Ninghe M. Cai, Justin M. Drogos, Julius P. A. Dewald, Netta Gurari

**Affiliations:** ^1^Department of Physical Therapy and Human Movement Sciences, Northwestern University, Chicago, IL, United States; ^2^Department of Biomedical Engineering, Northwestern University, Chicago, IL, United States

**Keywords:** perception, torque, stroke, evaluation methodology, mechatronics

## Abstract

**Background:** Successful execution of a task as simple as drinking from a cup and as complicated as cutting food with a fork and knife requires accurate perception of the torques that one generates in each arm. Prior studies have shown that individuals with hemiparetic stroke inaccurately judge their self-generated torques during bimanual tasks; yet, it remains unclear whether these individuals inaccurately judge their self-generated torques during unimanual tasks.

**Objective:** The goal of this work was to determine whether stroke affected how accurately individuals with stroke perceive their self-generated torques during a single-arm task.

**Methods:** Fifteen individuals with hemiparetic stroke and fifteen individuals without neurological impairments partook in this study. Participants generated a target torque about their testing elbow while receiving visual feedback, relaxed, and then matched the target torque about the same elbow without receiving feedback. This task was performed for two target torques (5 Nm, 25% of maximum voluntary torque), two movement directions (flexion, extension), and two arms (left, right).

**Results:** Clinical assessments indicate that eleven participants with stroke had kinaesthetic deficits and two had altered pressure sense; their motor impairments spanned from mild to severe. These participants matched torques at each elbow, for each target torque and movement direction, with a similar accuracy and precision to controls, regardless of the arm tested (*p* > 0.050).

**Conclusions:** These results indicate that an individual with sensorimotor deficits post-hemiparetic stroke may accurately judge the torques that they generate within each arm. Therefore, while survivors of a hemiparetic stroke may have deficits in accurately judging the torques they generate during bimanual tasks, such deficits do not appear to occur during unimanual tasks.

## 1. Introduction

Activities of daily living, including pulling open a drawer and cutting a fruit, require not only the correct generation, but also the accurate interpretation of movements (Cole and Sedgwick, 1992; Cole, 1995). Intact sensorimotor control is required for an individual to seamlessly carry out such actions. After a hemiparetic stroke, changes to force production and motor task execution of the paretic limb have been well-studied and documented (e.g., Hermsdörfer et al., 2003; Stinear et al., 2007; Lodha et al., 2010; Chang et al., 2013; Kang and Cauraugh, 2015). Changes in the paretic limb include weakness (Twitchell, 1951; Brunnström, 1970), hyperactive stretch reflexes (McPherson et al., 2018a,b), and loss of independent joint control (Dewald et al., 1995; Dewald and Beer, 2001; Sukal et al., 2007). Evidence also suggests that the non-paretic limb is affected after a stroke (Corkin et al., 1973; Carey and Matyas, 2011; Sainburg et al., 2016). However, the impact of a hemiparetic stroke on an individual's ability to perceive their self-generated forces in each limb has not been as extensively characterized.

Literature suggests that the perception of force is formed based on the processing of the descending motor commands and/or ascending sensory information (Proske and Allen, 2019). Previous research, using between-arms protocols, suggests that individuals with hemiparetic stroke perceive a reference torque about their elbow based mainly on the effort required to produce the torque (Bertrand et al., 2004; Mercier et al., 2004; Lodha et al., 2012; Yen and Li, 2015; van der Helm et al., 2017). Our earlier work revealed errors in matching torques between arms to the extent that an individual with hemiparetic stroke perceived their self-generated torque at their paretic arm as being seven times greater than at their non-paretic arm (Gurari et al., 2019). The deficit observed in a between-arms task could arise due to deficits within the paretic arm itself. Yet, previous studies have not addressed whether these individuals can accurately identify the torques that they generate during a single-arm task. This gap exists in our understanding of how a hemiparetic stroke impacts an individual's perception of torques they generate at their paretic limb. Therefore, we aimed to evaluate whether individuals with hemiparetic stroke can accurately match torques about their elbow within one arm.

Given the possibility that stroke impacts the non-paretic limb (Corkin et al., 1973; Carey and Matyas, 2011; Sainburg et al., 2016), we assessed the accuracy in matching self-generated torques about the elbow in both the paretic and non-paretic arms of individuals with hemiparetic stroke. We also compared their performance with that of similarly-aged individuals without neurological impairments, i.e., controls, who provided the baseline performance. Given the controversial impact of hand dominance on an individual's judgement of their self-generated torques (Weerakkody et al., 2003; Wang and Sainburg, 2004; Park et al., 2008; Sleimen-Malkoun et al., 2011; Wang et al., 2011; Adamo et al., 2012; Gueugnon et al., 2014; Scotland et al., 2014; van der Helm et al., 2017), we tested both the dominant and non-dominant arms of controls. Therefore, our goal was to characterize the impact of paresis from stroke, as well as hand dominance, on an individual's accuracy in judging their self-generated torques during a single-arm task. While studies support the notion that judging the torques that one generates during a between-arms task may be inaccurate, we hypothesize that this inaccurate judgement will not be apparent during a single-arm task. We hypothesize that individuals with sensorimotor deficits post-hemiparetic stroke will match torques within the same arm with a similar accuracy as controls.

## 2. Methods

The methods presented here were designed to resemble the methods used in our assessment of torque perception during a between-arms task so that we could interpret our results in light of those findings (van der Helm et al., 2017; Gurari et al., 2019). As such, we refer the reader to these previous publications for further information relevant to the design of this study.

### 2.1. Participants

The Northwestern University Institutional Review Board authorized human subject testing (STU00208205), and participants included in this study provided written informed consent. Inclusion criteria for all participants were the ability to understand and successfully execute the task and no serious pain or injury to the arm or peripheral nerves that could interfere with task execution and perception. Individuals with diabetes were excluded to avoid the possibility of participants having diabetic sensory neuropathies. For controls, they were required to be right-hand dominant and a similar age as the participants with hemiparetic stroke.

A licensed physical therapist (i.e., Dr. Justin M. Drogos) screened all participants with stroke to confirm their eligibility and to assess their motor and perceptual impairments via the upper-extremity Fugl-Meyer Motor Assessment (UE FMA) (Fügl-Meyer et al., 1975) and revised Nottingham Sensory Assessment (rNSA) (Lincoln et al., 1998; Stolk-Hornsveld et al., 2006), respectively. Additional inclusion criteria for participants with stroke were a single unilateral lesion of the brain located above the brainstem and not in the cerebellum; brain injury occurring >6 months prior to testing; no use of antispastic agents, e.g., Baclofen, in the past 6 months; and no neurological comorbidities.

### 2.2. Experimental Setup

**Figure 1:**
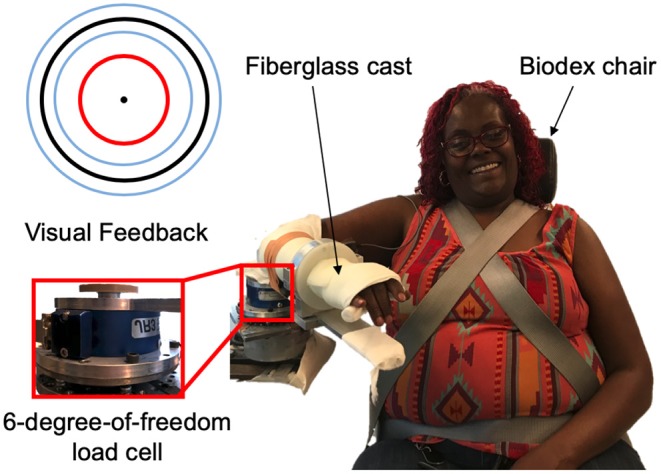
Experimental setup. (Right) An example individual interacting with the experimental setup. (Top, left) Visual feedback displayed on a monitor relayed information about the participant's applied torques about their elbow at the testing arm (red circle), target torque (black circle), and allowable range of applied torques (blue inner and outer circles). (Bottom, left) A multi-axis load cell captured the torques that the participant applied about their testing elbow.

The experimental setup, as shown in Figure 1, was comprised of a custom mechatronic system, monitor, speakers, and Biodex chair (System 3 Pro^TM^; Shirley, NY, USA). The mechatronic system included an isometric measurement device, which quantified the torques that the participant generated about their elbow joint using a six-degree-of-freedom force/torque sensor (JR3, Model: 45E15A 1000N; Woodland, CA, USA). The monitor provided the participant real-time visual feedback about the magnitude of their torques generated, and the speakers played aloud recorded audio cues instructing the participant which actions to execute. The Biodex chair restricted movements of the participant at their torso and waist. The software updated at 4 kHz, and trial-related data were stored at 1 kHz.

### 2.3. Experimental Protocol

The participant was requested to not exercise the day before and of testing to avoid muscle fatigue. At the beginning of the testing session, the participant sat with their torso and waist strapped to the Biodex chair. The participant's first testing arm was affixed to an isometric measurement device at 85° shoulder abduction, 40° shoulder flexion, and 90° elbow flexion.

Data collection began with quantifying the maximum voluntary torque (MVT) that the participant could generate about their elbow joint in flexion and extension. Next, we confirmed that motor impairments did not affect the ability of the participant to successfully match torques by verifying that the participant could generate and hold for 4 s 20 and 40% of their MVT in flexion and extension. Following, the participant completed the torque-matching trials. A target torque of 5 Nm was chosen as the fixed torque, and a target torque of 25% MVT was chosen as the percentage torque to address the strength differences in each arm of every participant. The four testing conditions were comprised of two directions (flexion, extension) at two target torques (fixed, percentage). For each of four testing conditions, the participant first became familiarized with the task by completing two practice trials. Then, the participant completed eight testing trials, which were used in the data analyses. Presentation order of the testing conditions was randomized across participants using a Latin square design.

These testing procedures were repeated for the opposite arm. The order of the arm first tested was randomized across participants.

### 2.4. Trial Timeline

**Figure 2:**
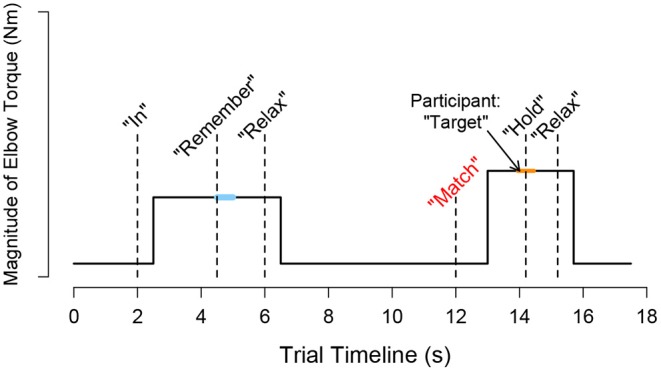
Schematic trial timeline for a torque matching task in flexion. During a trial, *i*, the participant followed automated audio and visual cues to generate a target torque with an arm and then to subjectively match the target torque using the same arm without visual feedback. The participant's self-generated torque at the target (τ_target_,_i_) was calculated as the average measured torque following 0.5 s after the audio cue “remember” played, as indicated by the light blue thick horizontal line. The participant's self-generated torque when indicating that the torque matched (τ_indicator, *i*_) was calculated as the average measured torque from 0.25 s before and after the “hold” sound played, as indicated by the orange thick horizontal line.

A visual depiction of the events occurring throughout a trial is provided in Figure 2. A trial began with the target torque visually depicted as a stationary blue circle on the monitor, which was situated in front of the participant. The acceptable range of torques that the participant could generate, i.e., a minimum of 80% and a maximum of 120% of the target torque, was visually depicted as the inner and outer light blue circles, respectively. To initiate the trial, an automated audio cue stated aloud “in” or “out” to the participant to indicate whether the direction of the target torque was in flexion or extension, respectively. The torque that the participant generated was visually conveyed by a red circle [Figure 1 (top, left)], whose diameter changed corresponding to the magnitude of the torque that the participant produced. The target torque was reached when the red circle, representing the magnitude of the participant's applied torque, was within the allowable range of applied torques (outlined by the inner and outer light blue circles) for 2 s. The audio cue, “remember,” then played to encourage the participant to remember and maintain the target torque for one additional second. Following, the participant was prompted by the automated audio cue to “relax.” After 6 s, “match” played to prompt the participant to generate a torque without receiving visual feedback on their self-generated torque. The participant verbally informed the experimenter “target” when the previously held target torque was perceived as matched. The audio cue, “hold,” played to instruct the participant to hold this indicator torque for 1 s. Following, “relax” played to signal the ending of the trial. The participant did not receive feedback about their torque-matching ability. The participant then briefly activated their antagonist muscles and relaxed for 20 s before starting the next trial to encourage quiescent muscle activity (McPherson et al., 2008).

## 3. Analysis

### 3.1. Strength Asymmetry Index

We used a strength asymmetry index for each direction (i.e., flexion, extension) to quantify the asymmetry in participant strength between arms. The strength asymmetry index was calculated in participants with stroke as the ratio of the MVT of the paretic arm divided by the MVT of the non-paretic arm, and in controls as the ratio of the MVT of the non-dominant arm divided by the MVT of the dominant arm. A strength asymmetry index of 1.0 indicates equal strength between arms. A strength asymmetry index <1.0 indicates that the paretic arm is weaker than the non-paretic arm for participants with stroke and the non-dominant arm is weaker than the dominant arm for controls. A strength asymmetry index >1.0 indicates that the paretic arm is stronger than the non-paretic arm for participants with stroke and the non-dominant arm is stronger than the dominant arm for controls.

### 3.2. Data Extraction

Data segments extracted and analyzed for each trial, *i*, are visually depicted in Figure 2. The measured target torque, τ_target,*i*_, was defined as the mean of 0.5 s of torque data when the participant held the target torque, and the measured indicator torque, τ_indicator,*i*_, as the mean of 0.5 s of torque data when the participant indicated that the torques were matched. The error in matching the target torque, τ_error,*i*_, was defined for each trial as the difference between the magnitude of the measured target torque and measured indicator torque, i.e., |τ_indicator,*i*_|−|τ_target,*i*_|. A positive and negative error indicated that the target torque was overshot and undershot, respectively. Three outcome measures were obtained for each testing condition (i.e., 2 target torques × 2 directions) of each arm of every participant. Constant error, or the mean error across the eight testing trials, indicated whether the participant generated too much or too little torque when matching the target torque. Absolute error, or the mean absolute error across the eight testing trials, indicated whether the participant accurately matched the target torque, regardless of whether too much or too little torque was generated. Variable error, or the standard deviation of the error across the eight testing trials, indicated whether the participant matched consistently using the same torque or was highly variable.

### 3.3. Statistical Testing

Analyses were run for each direction (i.e., flexion, extension), separately, to determine whether strength, i.e., MVT, depended on the arm tested. Additionally, analyses were run for each testing condition (i.e., 2 target torques × 2 directions), separately, to determine whether the three outcome measures (i.e., CE, AE, VE) depended on the arm tested. We used a linear-mixed effects model (Laird and Ware, 1982; Pinheiro and Bates, 2000) to run this analysis, where arm (i.e., dominant and non-dominant in controls; non-paretic and paretic in participants with stroke) was defined as a fixed effect and participant as a random effect. We ran an analysis of variance to identify significant differences and accounted for the multiple outcome measures using a Holm correction (Holm, 1979).

## 4. Results

### 4.1. Participants

Relevant information about each participant is provided in Table 1. Ten male and five female controls were tested. All participants were right-hand dominant (Oldfield, 1971) and had a mean ± standard deviation age of 57 ± 10 (range: 28–67).

**Table 1:** Participant information.

**Participant**	**Age (years)/**	**UE**	**rNSA elbow**	**rNSA elbow**	**Year(s)**	**Lesion**	**τ**_**MVT:flex**_	**τ**_**MVT:flex**_	**τ**_**MVT:ext**_	**τ**_**MVT:ext**_
	**Gender**	**FMA**	**kinaesthetic**	**pressure**	**since**	**location(s)**	**Par/Non-Dom**	**Non-Par/Dom**	**Par/Non-Dom**	**Non-Par/Dom**
		**score**	**sensation score**	**sensation score**	**stroke**	**(L: Left/R: Right)**	**arm (Nm)**	**arm (Nm)**	**arm (Nm)**	**arm (Nm)**
			**(Max of 3)**	**(Max of 2)**						
Stroke 1	53/M	17	2	2	31	NA	48	88	29	56
Stroke 2	49/M	31	2	2	15	R: Th, IC	48	77	25	48
Stroke 3	63/M	24	2	2	12	R: Th, IC, BG	39	84	11	62
Stroke 4	72/M	40	2	2	8	R: IC, Th, I	20	45	24	36
Stroke 5	48/M	18	2	1	11	R: Th, IC, BG	14	76	23	66
Stroke 6	66/F	25	2	2	11	L: BG, IC	16	19	15	12
Stroke 7	44/M	43	2	2	5	L: IC,BG,Th,F,P	57	70	34	47
Stroke 8	72/M	12	2	2	24	L: I, IC, Th, BG	23	76	16	69
Stroke 9	55/M	58	3	2	11	L: T-P	35	46	28	29
Stroke 10	29/F	20	3	2	1	L: F	17	26	13	17
Stroke 11	62/M	32	3	2	8	NA	47	65	40	51
Stroke 12	83/M	50	2	2	3	L: IC, I, P	52	63	37	46
Stroke 13	59/M	19	2	0	10	R: F, P	30	53	11	50
Stroke 14	62/M	45	3	2	5	R: IC	48	58	34	52
Stroke 15	60/M	57	2	2	8	L: IC, BG	50	72	35	51
Control 1	62/M	–	–	–	–	–	90	97	69	71
Control 2	44/M	–	–	–	–	–	73	79	52	52
Control 3	60/M	–	–	–	–	–	62	66	35	42
Control 4	63/M	–	–	–	–	–	90	94	42	66
Control 5	64/F	–	–	–	–	–	46	42	32	31
Control 6	62/M	–	–	–	–	–	78	80	75	65
Control 7	55/M	–	–	–	–	–	62	75	50	51
Control 8	61/M	–	–	–	–	–	82	99	59	58
Control 9	28/M	–	–	–	–	–	74	61	54	42
Control 10	67/M	–	–	–	–	–	33	34	23	27
Control 11	60/F	–	–	–	–	–	35	37	22	24
Control 12	61/F	–	–	–	–	–	37	36	23	26
Control 13	50/F	–	–	–	–	–	39	42	27	28
Control 14	64/F	–	–	–	–	–	44	39	28	31
Control 15	61/M	–	–	–	–	–	62	65	56	55

*This table summarizes relevant clinical and experimental information about each participant. M, male; F, female; UE FMA, upper-extremity Fugl-Meyer Motor Assessment; rNSA, revised Nottingham Sensory Assessment; Max, maximum; τ_MVT: flex_, maximum voluntary torque in elbow flexion; τ_MVT: ext_, maximum voluntary torque in elbow extension; Dom, dominant; Non-Dom, non-dominant; Par, paretic; Non-Par, non-paretic; NA, not available; -, data not relevant to the participant; Th, thalamus; IC, internal capsule; BG, basal ganglia; F, frontal; P, parietal; O, occipital; T, temporal; T-P, tempo-parietal; I, insula*.

Thirteen male and two female participants with hemiparetic stroke were tested. Twelve were right-hand dominant (Oldfield, 1971), and nine had a right-arm paresis. Participants with stroke had a mean ± standard deviation age of 57 ± 11 (range: 29–83) and were 11 ± 8 years since their stroke (range: 1–31).

Sensorimotor deficits of participants with stroke, as determined by the UE FMA and rNSA, are reported in Table 1. Participants with stroke had an UE FMA score that spanned 17–57 (μ±σ: 33 ± 15), representing motor impairments ranging from mild to severe. Based on their rNSA elbow kinaesthetic sensation score, eleven of the fifteen participants with stroke had deficits in identifying the location of their paretic limb in space. Based on their rNSA elbow pressure sensation score, out of the fifteen participants with stroke, only two participants had altered pressure sensation in their paretic arm.

### 4.2. Sensorimotor Control

#### 4.2.1. Strength

The strength among participants in flexion and extension is summarized in Table 1. Participants with stroke generated less MVT in their paretic arm than their non-paretic arm (flexion: p<0.001; extension: *p* < 0.001), the non-dominant arm of controls (flexion: *p* = 0.004; extension: *p* = 0.003), and the dominant arm of controls (flexion: *p* = 0.001; extension: *p* = 0.004). Comparing the non-paretic arm of participants with stroke with either arm of controls, our analyses did not reveal significant differences in the MVT generated in either flexion (non-dominant: *p* = 0.838; dominant: *p* = 0.515) or extension (non-dominant: *p* = 0.942; dominant: *p* = 0.830). For controls, the MVT generated by their dominant and non-dominant arms did not significantly differ in either flexion (*p* = 0.427) or extension (*p* = 0.839).

**Figure 3:**
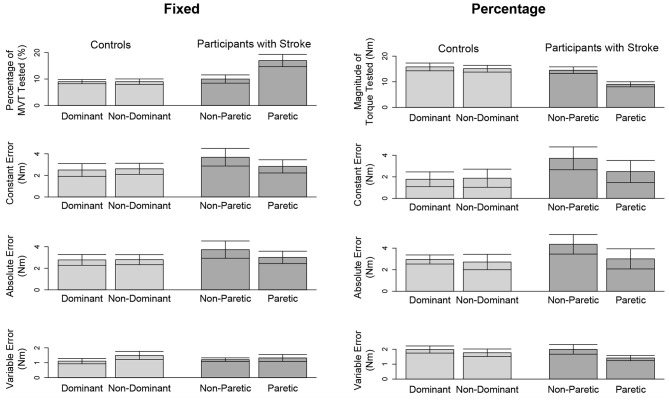
Outcome measures when matching in flexion. Mean and standard error of participants' tested target torques, constant errors, absolute errors, and variable errors when matching in flexion a target torque of **(left)** 5 Nm and **(right)** 25% of their testing arm's MVT.

**Figure 4:**
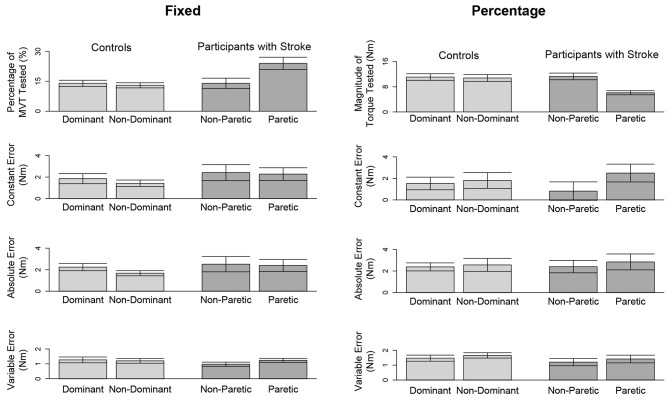
Outcome measures when matching in extension. Mean and standard error of participants' tested target torques, constant errors, absolute errors, and variable errors when matching in extension a target torque of **(left)** 5 Nm and **(right)** 25% of their testing arm's MVT.

In Figure 3 (top, left) and Figure 4 (top, left), we identify the percentage of MVT at which participants were tested during the fixed task when the target torque was 5 Nm. When matching in flexion, the 5 Nm target torque corresponded to a mean ± standard deviation of 8.8 ± 3.4 and 9.2 ± 3.4% of the MVT of the dominant and non-dominant arm in controls, and 10.0 ± 5.6 and 17.1 ± 9.5% of the non-paretic and paretic arm in participants with stroke. When matching in extension, the 5 Nm target torque corresponded to 12.7 ± 4.6 and 13.4 ± 6.0% of the MVT of the dominant and non-dominant arm in controls, and 13.4 ± 7.8 and 24.0 ± 12.2% of the non-paretic and paretic arm in participants with stroke.

In Figure 3 (top, right) and Figure 4 (top, right), we indicate the magnitude of torque at which participants were tested when the target torque was 25% of their testing arm's MVT. 25% MVT in flexion corresponded to a mean ± standard deviation of 16.0 ± 5.4 and 15.2 ± 5.2 Nm at the dominant and non-dominant arm of controls, and 14.8 ± 4.9 and 9.4 ± 4.4 Nm at the non-paretic and paretic arm of participants with stroke. 25% MVT in extension corresponded to a range of 11.0 ± 3.6 and 11.2 ± 4.7 Nm at the dominant and non-dominant arm of controls, and 11.3 ± 4.0 and 6.5 ± 3.0 Nm at the non-paretic and paretic arm of participants with stroke.

#### 4.2.2. Strength Asymmetry Between Arms

The strength asymmetry index for controls ranged from 0.83 to 1.21 (μ ± σ: 0.98 ± 0.10) and 0.64 to 1.29 (μ ± σ: 0.96 ± 0.15) in flexion and extension, respectively. For participants with stroke, the strength asymmetry index in flexion ranged from 0.18 to 0.84 (μ ± σ: 0.62 ± 0.20) and in extension ranged from 0.18 to 1.25 (μ ± σ: 0.62 ± 0.29).

### 4.3. Matching of Torques

#### 4.3.1. Flexion

The accuracy and precision of participants when matching each target torque are reported in Table 2 and Figure 3. Among the three outcome measures quantifying torque-matching errors, no differences between the dominant and non-dominant arm of the controls and non-paretic and paretic arm of the participants with stroke were found when matching a torque in flexion of 5Nm (CE: *p* = 0.307; AE: *p* = 0.422; VE: *p* = 0.383) or 25% MVT (CE: *p* = 0.410; AE: *p* = 0.271; VE: *p* = 0.160). Additionally, we acknowledge that the lesioned hemisphere and arm dominance of the participants with stroke were heterogeneous. Our analyses, however, did not reveal differences in torque-matching errors depending on arm dominance or lesioned hemisphere (*p* > 0.050).

**Table 2:** Statistical results when matching in flexion.

**Target Torque**	**Group**	**Arm**	****μ**_**torque**_**	**CE**	**AE**	**VE**
			**(Nm)**	**(Nm)**	**(Nm)**	**(Nm)**
	Controls	Dominant	5.1 ± 0.3	2.5 ± 2.3	2.8 ± 1.9	1.1 ± 0.7
			(4.5, 5.5)	(−1.6, 6.8)	(0.5, 6.8)	(0.4, 3.0)
5 Nm		Non-dominant	5.1 ± 0.2	2.6 ± 2.0	2.8 ± 1.8	1.5 ± 1.1
			(4.7, 5.4)	(−0.9, 7.5)	(0.7, 7.5)	(0.5, 4.5)
		Non-paretic	5.0 ± 0.2	3.7 ± 3.1	3.7 ± 3.1	1.2 ± 0.5
	Participants		(4.6, 5.3)	(−0.2, 11.2)	(0.4, 11.2)	(0.4, 2.3)
	with stroke	Paretic	4.8 ± 0.3	2.8 ± 2.3	3.0 ± 2.2	1.3 ± 0.9
			(4.4, 5.6)	(0.1, 5.9)	(0.4, 5.9)	(0.4, 3.9)
	Controls	Dominant	16.1 ± 5.6	1.8 ± 2.6	2.9 ± 1.6	2.0 ± 0.9
			(8.7, 23.8)	(−3.6, 6.3)	(0.6, 6.3)	(0.8, 3.7)
25%MVT		Non-dominant	14.9 ± 5.2	1.9 ± 3.3	2.7 ± 2.8	1.8 ± 1.0
			(8.2, 24.7)	(−1.3, 11.0)	(0.6, 11.0)	(0.7, 4.0)
		Non-paretic	14.5 ± 4.8	3.7 ± 4.1	4.4 ± 3.5	2.0 ± 1.3
	Participants		(4.7, 19.9)	(−1.7, 10.7)	(0.5, 10.9)	(0.3, 5.4)
	with stroke	Paretic	9.0 ± 4.5	2.5 ± 4.0	3.0 ± 3.6	1.4 ± 0.7
			(3.3, 18.8)	(−0.9, 13.5)	(0.5, 13.5)	(0.3, 2.3)

*Mean ± standard deviation and range (minimum, maximum) are reported for the measured target torque and every outcome measure. μ_torque_, mean magnitude of measured target torque; CE, constant error; AE, absolute error; VE, variable error*.

#### 4.3.2. Extension

Due to a lack of steady control, Stroke 6 was unable to hold their self-generated torques in extension within 80 and 120% of the target torque for 2 s when using their paretic arm. Therefore, this participant's data were not included in the following analyses. The accuracy and precision of the remaining 14 participants when matching each target torque are reported in Table 3 and Figure 4. Among the three outcome measures quantifying the torque-matching errors, no differences between the dominant and non-dominant arm of the controls and non-paretic and paretic arm of the participants with stroke were found when matching a torque in extension of 5Nm (CE: *p* = 0.533; AE: *p* = 0.297; VE: *p* = 0.531) or 25% MVT (CE: *p* = 0.367; AE: *p* = 0.892; VE: *p* = 0.522). Our analyses did not find any significant differences in torque-matching errors depending on the lesioned hemisphere and arm dominance of the participants with stroke (*p* > 0.050).

**Table 3:** Statistical results when matching in extension.

**Target Torque**	**Group**	**Arm**	****μ**_**torque**_**	**CE**	**AE**	**VE**
			**(Nm)**	**(Nm)**	**(Nm)**	**(Nm)**
	Controls	Dominant	5.0 ± 0.3	1.9 ± 1.8	2.2 ± 1.3	1.3 ± 0.8
			(4.5, 5.5)	(−1.4, 5.2)	(0.4, 5.2)	(0.5, 3.2)
5 Nm		Non-dominant	4.9 ± 0.2	1.4 ± 1.2	1.7 ± 1.0	1.2 ± 0.6
			(4.5, 5.4)	(−0.7, 3.2)	(0.4, 3.2)	(0.5, 2.3)
		Non-paretic	4.9 ± 0.2	2.4 ± 2.7	2.5 ± 2.6	1.0 ± 0.5
	Participants		(4.5, 5.4)	(−0.3, 8.0)	(0.3, 8.1)	(0.4, 2.4)
	with stroke	Paretic	4.8 ± 0.3	2.3 ± 2.2	2.4 ± 2.1	1.2 ± 0.5
			(4.4, 5.5)	(0.2, 7.9)	(0.5, 7.9)	(0.6, 2.4)
	Controls	Dominant	10.9 ± 3.7	1.5 ± 2.3	2.4 ± 1.5	1.5 ± 0.8
			(6.0, 17.5)	(−1.9, 5.5)	(0.3, 5.5)	(0.3, 3.3)
25%MVT		Non-dominant	10.9 ± 4.5	1.8 ± 2.8	2.6 ± 2.3	1.7 ± 0.7
			(5.4, 17.8)	(−1.6, 8.6)	(0.4, 8.6)	(0.5, 2.8)
		Non-paretic	11.3 ± 3.8	0.8 ± 3.0	2.4 ± 2.0	1.2 ± 0.9
	Participants		(3.9, 17.5)	(−3.3, 6.9)	(−0.2, 6.9)	(0.2, 2.9)
	with stroke	Paretic	6.1 ± 3.3	2.5 ± 2.9	2.8 ± 2.6	1.4 ± 0.9
			(2.4, 13.6)	(−1.4, 8.5)	(0.5, 8.5)	(0.4, 3.4)

*Mean ± standard deviation and range (minimum, maximum) are reported for the measured target torque and every outcome measure. μ_torque_, mean magnitude of measured target torque; CE, constant error; AE, absolute error; VE, variable error*.

## 5. Discussion

This work investigated whether individuals with hemiparetic stroke could accurately identify the torques that they generated about each elbow joint, independently. The main finding is that our tested participants with stroke could judge sub-maximal isometric torques that they generated within each limb with a similar accuracy and precision as our tested participants without neurological impairments.

### 5.1. Controls

We included the results of controls to quantify baseline performance when referencing a sub-maximal torque about each elbow. Findings based on the controls indicate that the accuracy and precision in matching sub-maximal torques about a single elbow were not impacted by the arm tested (i.e., dominant, non-dominant), regardless of the direction (i.e., flexion, extension) and magnitude (i.e., 5 Nm, 25% MVT) of the torque. Several previous studies have suggested a potential effect of arm dominance on the utilization of proprioceptive feedback (Scotland et al., 2014), especially for position control (Goble and Brown, 2007; Goble et al., 2009). However, in terms of accuracy and precision in matching a torque, we did not observe an effect of arm dominance. A possible reason for not observing a significant effect is the relatively small sample size of the controls. Even so, the magnitude of torque-matching errors (i.e., absolute error) between the dominant and non-dominant arm in controls did not differ more than 0.5 Nm, regardless of the torque magnitude and direction. Therefore, while collecting data from additional controls might lead to a significant difference in torque-matching errors, the current data indicate that the effect size, or difference, would be quite small.

### 5.2. Participants With Stroke

To begin, we highlight that the tested participants with stroke had motor impairments, according to their UE FMA scores and paretic limb weakness, that ranged from mild to severe. Even so, we confirmed that their ability to match torques was not influenced by their motor impairments. This was achieved by verifying prior to testing that the participant could generate and maintain a target torque for at least 4 s.

We compared the torque-matching ability of the participants post-stroke with that of the controls to identify potential deficits in judging torques within a single arm. Previous studies suggest that errors in matching torques between arms are associated with the relative weakness of the paretic arm (Bertrand et al., 2004; Mercier et al., 2004; Yen and Li, 2015). In our study, participants with stroke had varying degrees of hemiparesis, represented by the strength asymmetry index ranging from 0.18 to 1.25. Nonetheless, our participants with hemiparetic stroke, when matching torques within a single arm, had magnitudes of errors considerably less than those in our group's previous studies in which individuals with stroke were requested to match torques between arms (e.g., van der Helm et al., 2017; Gurari et al., 2019). For comparison, in this study, when matching a fixed torque of 5 Nm within a single arm, torque-matching errors reached upwards of 7.9 Nm at the paretic arm of our participants with stroke and 6.8 Nm at the dominant arm of controls. Previous testing on matching a fixed torque of 5 Nm between-arms revealed errors that reached upwards of 18.5 Nm in a similar group of participants with stroke and 7.6 Nm in controls (Gurari et al., 2019). Therefore, even though our participants with stroke were hemiparetic, and consequently, might exhibit deficits in matching torques between-arms, our findings indicate they could judge their self-generated torques within each arm similarly to controls.

The perception of forces in individuals without neurological impairments is generally thought to have both a peripheral origin from mechanoreceptors (Jones and Piateski, 2006; Luu et al., 2011), such as the Golgi tendon organ (Roland and Ladegaard-Pedersen, 1977; Jami, 1992), and a central origin from signals related to motor commands (McCloskey et al., 1974; Jones and Hunter, 1983; Scotland et al., 2014). However, the exact neural mechanism underlying the perception of forces has yet to be elucidated and remains an area of debate (Proske and Allen, 2019). Results of our study suggest that individuals with hemiparetic stroke were able to reproduce a rotational force, i.e., torque, about their elbow using the same arm, with a comparable accuracy and precision as individuals without neurological impairments. It is possible that they utilized centrally generated signals, such as an efference copy, when matching using the same arm, even if the signal was erroneous. It is also possible that participants with stroke relied on afferent feedback, albeit potentially erroneous, arising from the mechanoreceptors to match an elbow torque in the same arm. As such, our study does not provide insight about potential neural mechanisms used when judging torques in individuals post-stroke. Nevertheless, this study aids in our current understanding of force perception post-stroke by demonstrating that information used to match sub-maximal torques within a single arm is reliable enough to allow an accurate and precise reproduction of previously generated torques in individuals with hemiparetic stroke.

### 5.3. Limitations

Our experimental design required individuals to control a steady torque for 3 s to successfully execute a torque-matching trial. For individuals with stroke, due to weakness or lack of control of their self-generated torques, it could be difficult to maintain a constant torque for 3 s. We had to exclude four individuals with stroke during screening. Hence, findings from this study are unable to address torque-matching ability within a single arm of individuals with hemiparetic stroke who present such motor impairments.

Additionally, daily activities may involve a generation of torques lasting longer than 3 s, and maintaining a torque for a longer duration could result in increased variability of the torque production for individuals with stroke (Lodha et al., 2013; Kang and Cauraugh, 2015). An increase in torque production variability can negatively affect how precisely a torque is perceived (Gurari et al., 2017). As such, a limitation of this work is that it does not assess how accurately and precisely individuals post-hemiparetic stroke can identify torques that are maintained for >3 s. However, fatigue would pose an experimental challenge, particularly for participants with stroke, if participants are required to hold target torques for a longer duration.

Moreover, our recruitment did not yield participants with stroke who were clinically assessed with severe sensory impairments. Therefore, it is not clear whether findings from this study can be extended to populations who are identified as having more severe sensory deficits.

## 6. Conclusions and Future Directions

Current evidence suggests that, when matching a sub-maximal torque about the elbow within a single arm, individuals with hemiparetic stroke can achieve a similar accuracy and precision as individuals with neurological impairments. This result highlights that, even though individuals with hemiparetic stroke might have deficits in matching torques between-arms as indicated by previous studies (Bertrand et al., 2004; Yen and Li, 2015; Gurari et al., 2019), they may reliably reproduce previously generated torques within a single arm. Future work plans to expand this line of research to address how accurately individuals with hemiparetic stroke perceive their self-generated torques during multi-degree-of-freedom isometric tasks. Participants in this study were asked to generate a one-degree-of-freedom isometric torque, i.e., a torque about their elbow joint, which has limited applications in the real world. Most activities of daily living involve the simultaneous generation of torques at numerous joints. Furthermore, the literature highlights the challenges that individuals with hemiparetic stroke face in controlling independent joint movements (Dewald et al., 1995; Dewald and Beer, 2001; Sukal et al., 2007). Therefore, future work can address the effect of multi-joint isometric tasks on the accuracy of individuals with hemiparetic stroke in judging their self-generated torques.

## Data Availability Statement

The raw data supporting the conclusions of this manuscript will be made available by the authors, without undue reservation, to any qualified researcher.

## Ethics Statement

The studies involving human participants were reviewed and approved by Northwestern University Institutional Review Board. The patients/participants provided their written informed consent to participate in this study. Written informed consent was obtained from the individual(s) for the publication of any potentially identifiable images or data included in this article.

## Author Contributions

NG, NC, JMD, and JPAD: study design and manuscript editing. NG, NC, and JMD: data acquisition. NG and NC: data analysis, data interpretation, and manuscript preparation.

### Conflict of Interest

The authors declare that the research was conducted in the absence of any commercial or financial relationships that could be construed as a potential conflict of interest.
